# *Leptospira* Serovars for Diagnosis of Leptospirosis in Humans and Animals in Africa: Common *Leptospira* Isolates and Reservoir Hosts

**DOI:** 10.1371/journal.pntd.0004251

**Published:** 2015-12-01

**Authors:** Georgies F. Mgode, Robert S. Machang’u, Ginethon G. Mhamphi, Abdul Katakweba, Loth S. Mulungu, Lies Durnez, Herwig Leirs, Rudy A. Hartskeerl, Steven R. Belmain

**Affiliations:** 1 Pest Management Centre, Sokoine University of Agriculture, Morogoro, Tanzania; 2 Department of Biomedical Sciences, Institute of Tropical Medicine, Antwerp, Belgium; 3 Evolutionary Ecology Group, University of Antwerp, Antwerpen, Belgium; 4 Royal Tropical Institute (KIT), KIT Biomedical Research, WHO/FAO/OIE and National Collaborating Centre for Reference and Research on Leptospirosis, Amsterdam, The Netherlands; 5 Natural Resources Institute, University of Greenwich, United Kingdom; Swiss Tropical and Public Health Institute, SWITZERLAND

## Abstract

The burden of leptospirosis in humans and animals in Africa is higher than that reported from other parts of the world. However, the disease is not routinely diagnosed in the continent. One of major factors limiting diagnosis is the poor availability of live isolates of locally circulating *Leptospira* serovars for inclusion in the antigen panel of the gold standard microscopic agglutination test (MAT) for detecting antibodies against leptospirosis. To gain insight in *Leptospira* serovars and their natural hosts occurring in Tanzania, concomitantly enabling the improvement of the MAT by inclusion of fresh local isolates, a total of 52 *Leptospira* isolates were obtained from fresh urine and kidney homogenates, collected between 1996 and 2006 from small mammals, cattle and pigs. Isolates were identified by serogrouping, cross agglutination absorption test (CAAT), and molecular typing. Common *Leptospira* serovars with their respective animal hosts were: Sokoine (cattle and rodents); Kenya (rodents and shrews); Mwogolo (rodents); Lora (rodents); Qunjian (rodent); serogroup Grippotyphosa (cattle); and an unknown serogroup from pigs. Inclusion of local serovars particularly serovar Sokoine in MAT revealed a 10-fold increase in leptospirosis prevalence in Tanzania from 1.9% to 16.9% in rodents and 0.26% to 10.75% in humans. This indicates that local serovars are useful for diagnosis of human and animal leptospirosis in Tanzania and other African countries.

## Author Summary

Leptospirosis disease is widespread in humans and broad range of animal species in Africa. However, leptospirosis is highly neglected and not extensively taught in both medical and veterinary schools almost across Africa. Availability of live leptospires isolated from Africa for use in its diagnosis by the gold standard microscopic agglutination test (MAT) is also a major problem. This study reports on local *Leptospira* serovars and their natural hosts consisting of different animal species, for inclusion in diagnosis of leptospirosis in Africa. A total of 52 *Leptospira* isolates were obtained from fresh urine and kidneys. African giant pouched rats (*Cricetomys* sp.) and insectivore shrew species (*Crocidura* sp.) had the highest leptospires isolation success. These were identified by serogrouping, cross-agglutination absorption test and molecular typing. Common *Leptospira* serovars with their respective animals were: serovar Sokoine (cattle and rodents); Kenya (rodents and shrews); Mwogolo (rodents); Lora (rodents); Canicola (rodents); Grippotyphosa (cattle); and an unknown serogroup from pigs. Inclusion of local serovar Sokoine in serodiagnosis revealed a 10-fold increase in leptospirosis prevalence from 1.9% to 16.9% in rodents and 0.26% to 10.75% in humans. Future serodiagnosis of leptospirosis in Africa should include these serovars and serovar Hardjo, Hebdomadis, Pomona and other local isolates.

## Introduction

Leptospirosis is an understudied zoonotic disease in Tanzania and across Africa. Limited reports show a high prevalence of leptospirosis in animals and humans with Africa presenting a major burden globally [1]. The highest median annual incidence of leptospirosis is in Africa standing at 95.5 per 100,000 people. Africa is followed by Western Pacific (66.4), the Americas (12.5), South-East Asia (4.8) and Europe (0.5) [2]. In Africa, leptospirosis has been reported in almost all geographic zones. Despite its high burden, leptospirosis is not routinely diagnosed in African hospitals. Awareness of this disease is also generally lacking among health providers, medical personnel and the general public including high-risk populations such as abattoir workers and animal handlers (Mgode, *personal observation*). One of the major factors limiting diagnosis of leptospirosis in Africa is the need for leptospire isolation to discover the local circulating serovars that are needed for inclusion in the microscopic agglutination test (MAT). MAT requires various live *Leptospira* serovars as antigens to detect infections caused by different serovars belonging to different serogroups [3, 4].

Apart from serological studies, there are relatively few studies on isolation and identification of *Leptospira* pathogens in Africa. However, a number of *Leptospira* serovars have been isolated in some African countries, namely South Africa [5, 6], Zimbabwe [7, 8], Congo DRC [9], Kenya [10, 11], Madagascar and other Indian ocean Islands [12, 13], Nigeria and Ghana [9, 14, 15], Egypt [16] and Tanzania. In Tanzania, leptospirosis is widely reported in wild small mammals, domestic animals and humans [17–22]. Despite these reports, awareness of this disease is still lacking and there is an urgent quest for gathering sufficient data on leptospirosis for promoting awareness. The objectives of this study were, therefore, to determine *Leptospira* serovars occurring in Tanzania and their host animals. This knowledge will help to rationally design control and prevention measures and contribute to an improved MAT for diagnostic and prevalence study purposes in Tanzania and potentially other East African countries.

## Materials and Methods

### Domestic and wild animal sampling

Cattle brought at Morogoro municipal abattoir and pigs slaughtered at two main informal slaughterhouse sites also in Morogoro were randomly sampled for this study. Sheep, goats, dogs and cats were also sampled from different localities in Morogoro. Trapping of rodent species and insectivore shrews was stratified whereby rodents were trapped in selected localities representing all geographic areas of Morogoro town including peri-urban areas, urban areas, inside houses, around houses (outdoors), fallow land, swampy areas, inside and around markets, inside and around the abattoir. Sherman live traps baited with peanut butter mixed with maize bran were used to trap small rodents and insectivores (shrew) species. Larger rodents particularly the African giant pouched rats were trapped in same localities as small rodents using Havahart traps baited with fresh maize cobs. The traps were set for three consecutive nights at each site. All captured animals were identified to genus level and geographic coordinates of the collection site were recorded using GPS.

### Human subjects

Blood samples for serological determination of leptospirosis by microscopic agglutination test were obtained from patients providing blood in various hospitals in Morogoro for other tests such as diagnosis of typhoid fever. Participants were orally informed that their samples would be anonymously tested for leptospirosis. 3–4 ml of blood was obtained and a drop was inoculated into fresh medium for isolation of leptospires. The remaining blood was centrifuged to obtain serum for serological test. Urine from abattoir workers was collected into sterile universal bottles and transported to the laboratory for inoculation of drops into sterile EMJH medium.

### Isolation of leptospires from animals

The isolation of leptospires from animal hosts in Tanzania started in 1996 and is ongoing. Isolation and identification of leptospires have been carried out in humans, domestic animals including cattle and pigs, and feral and (semi) domestic small mammals collected in natural landscapes, agricultural fields, in rural settlements and in urban areas. Wild and domestic animal collection and handling followed the guidelines of the American Society of Mammalogists [23]. Urine specimens were aseptically collected from animals anesthetised using di-ethyl ether. The urine was collected using sterile syringes and needles. Urine sampling was also done from cattle at slaughterhouse whereby the urinary bladder were taken out slaughtered animals and the neck of the bladder was tightly closed with fishing line (thread) to prevent spillage of urine during transportation to the laboratory. A drop of fresh urine was aseptically taken from the bladder using sterile syringe and needle and inoculated into a tube containing sterile Leptospira Ellinghausen and McCullough, modified by Johnson and Harris (EMJH) culture medium containing 5-Fluorouracil selective inhibitor. Kidney specimens were obtained after swabbing the sacrificed animal abdomen with 70% ethanol and dissecting using pair of sterile scissors and forceps. Smaller kidneys were put into sterile glass tube containing sterile phosphate buffered saline (pH 7.0) and homogenised using sterile glass rods and or sterile glass Pasteur pipettes. Larger kidneys were macerated to obtain cross-sectional pieces which were homogenized. A drop of kidney homogenate was aseptically inoculated into EMJH medium as previously described [24]. Cultures were incubated at 30°C for up to 8 weeks and examined weekly for *Leptospira* growth. Isolation of leptospires from fish species was not conducted in the present study whereas serological testing using putative prevalent serovars indicated potential to provide first insight on leptospirosis in fishes from this area.

### Identification of *Leptospira* isolates

Initially, five *Leptospira* isolates which were the first isolates from Tanzania of which three were from cattle and two from rodents were identified and enabled identification of many other isolates among the 52 reported in this study. *Leptospira* isolates SH9 and SH25 from the African giant pouched rats (*Cricetomys* sp.) and RM1 from cattle were subjected to standard taxonomical analyses recommended by the International Committee on Systematics of Prokaryotes: Subcommittee on the Taxonomy of Leptospiraceae. These were identified as serovar Kenya [18] and serovar Sokoine [19]. This enabled assigning other serologically and genetically identical isolates among the 52 isolates to serovar Kenya and Sokoine [25]. Isolates coded RM4 and RM7 were subjected to serological typing using serogrouping using reference rabbit serum and monoclonal antibodies. Multilocus sequence typing was also employed to identify sequent isolates from wild rodents and insectivores (*Crocidura* spp.) from Tanzania through determination of their genetic relatedness with known serovars. This enhanced assigning subsequent isolates to serovars Mwogolo, Lora, Kenya, Qunjian (Canicola) and Sokoine [25]. The typical identification procedures were as follows:

### Serogrouping

Selected *Leptospira* isolates coded RM1, RM4 and RM7 from cattle and SH9 and SH25 from the African giant pouched rats (*Cricetomys* sp.) were grown in EMJH medium for 5–7 days at 30°C. Fully-grown cultures with density of 3x10^8^ leptospires per ml were checked for purity under-darkfield microscopy before injecting into pair of healthy and leptospirosis-free animals to produce antiserum for serological identification of the isolates [26]. Animals were handled in compliance with the “Animal Research: Reporting In Vivo Experiments” (ARRIVE guidelines) and the Helsinki Declaration [27, 28]. The isolates were thereafter reacted with reference rabbit antiserum for common *Leptospira* serogroups. MAT was performed as previously described [3]. Findings of this prelinanry reaction were useful in selecting reacting serogroups for further identification of new isolates by monoclonal antibodies and cross-agglutination absorption test (CAAT) which uses antiserum produced using unknown (new isolates) and reference serovars as described below.

### Serotyping with monoclonal antibodies

MAT sets of specific monoclonal antibodies belonging to candidate *Leptospira* serogroups were used to determine isolate affiliation. Two isolates coded RM4 and RM7 were subjected to this approach employing the following monoclonal antibodies for serogroup Grippotyphosa: F71C2-4, F71C3-3, F71C9-4, F71C13-4, F71C16-6, F71C17-5, F164C1-1, F165C1-4, F165C2-1, F165C3-4, F165C7-5, F165C8-3 and F165C12-4. Other monoclonal antibodies used were of serovar Butembo serogroup Autumnalis: F43C9-5, F46C1-1, F46C2-4, F46C4-1, F46C5-1, F46C9-1, F46C10-1, F48C1-3, F48C3-3, F48C6-4, F58C1-2, F58C2-3 and F61C7-1. The monoclonal antibodies were provided by the WHO/FAO/OIE Collaborating Centre for Reference and Research on Leptospirosis, at the Royal Tropical Institute, Amsterdam, The Netherlands, which offers varieties of reference leptospirosis research materials worldwide [29].

### Molecular typing

*Leptospira* isolates coded SH9 and SH25 were also subjected to DNA fingerprinting described by Zuerner and Bolin [30, 31] and had identical DNA pattern of serovar Kenya [18]. Isolates coded TE 0826 and TE 0845 have been previously identified using multilocus sequence typing as identical to serovar Mwogolo serogroup Icterohaemorrhagiae; isolates TE 1992, TE 2324, TE 2364 and TE 2366 as serovar Lora serogroup Australis; and isolate coded TE 2980 as serovar Qunjian [25]. Additionally, 3 isolates were previously identified using multilocus sequence typing as serovar Sokoine, and 11 isolates as serovar Kenya [25].

### Cross agglutination absorption test (CAAT)

Further identification of *Leptospira* isolates was achieved using the gold standard test for identification and taxonomy of *Leptospira* serovars known as cross agglutination absorption test (CAAT). Briefly, isolates are identified as different serovars if more than 10% of homologous titre remains in one of the test isolates after cross-absorption with sufficient amount of heterologous antigen. This means that 0–10% difference in antibodies remaining following absorption represents strains belonging to same serovar [26]. *Leptospira* isolate is grown into EMJH medium to density of 3x10^8^ leptospires per ml for inoculation into laboratory rabbits to produce antibodies (antisera) against the isolate. Antiserum is harvested from rabbit and a titre of 1:5120 is determined by MAT using formalin killed homologous isolate. Antiserum with higher titre than 1:5120 is diluted with phosphate buffered saline. The test isolate cultured for 5–7 days in EMJH medium reaching a density of 3x10^8^ leptospires per ml is killed by mixing with formalin (final concentration of 0.5%) and incubating at room temperature for 1 hour. Formalized culture is divided into 5, 10 and 20 ml and centrifuged in refrigerated centrifuge at 10,000 rpm x *g* for 30 minutes to obtained sediment. The culture sediment is air dried and resuspended into PBS-formalin diluted antiserum. The suspension is incubated at 30°C overnight and thereafter centrifuged at 10,000 rpm x *g* for 30 minutes to obtain the supernatant which is the now the absorbed serum for MAT checking for absorption levels (under or over absorption) using live and killed antigen similar to the absorbing antigen. The supernatant with titre of 1:40–1:80 that is lower than 1% of homologous titre for the same antiserum will be chosen for control MAT with unabsorbed diluted reference antiserum with live and killed homologous antigen; and MAT with absorbed serum with live and killed homologous reference antigen. Subsequently, the produced antiserum against unknown isolate is absorbed with all positive *Leptospira* serovars to determine titres remaining after absorption. Detailed procedures of cross-agglutination absorption test have been described by Hartskeerl and co-workers [32]. *Leptospira* isolates coded SH9 and SH25 from the African giant pouched rats and isolate coded RM1 from cattle in Tanzania were identified as serovar Kenya and serovar Sokoine by this method [18, 19].

### Isolates deposit in culture collection

The reference *Leptospira* serovar Sokoine strain RM1 which is a new serovar described in 2006 has been deposited in *Leptospira* culture collection at the WHO/FAO/OIE Collaborating Centre for Reference and Research on Leptospirosis of the Royal Tropical Institute, Amsterdam, The Netherlands, and the WHO Collaboration Centre for Diagnosis, Reference, Research and Training in Leptospirosis, Port Blair, Andaman and Nicobar Islands, India [19]. Serovar Kenya strain SH9 and SH25 and as well as serovars Grippotyphosa strain RM4 and RM7, Mwogolo, Lora and Canicola reported in this study have also been deposited in the *Leptospira* culture collection of the Royal Tropical Institute, Amsterdam, The Netherlands. The isolates are also maintained in the culture collection of the Pest Management Centre, Sokoine University of Agriculture, Morogoro, Tanzania.

### Distribution of *Leptospira* serovars in different wild and domestic mammals, fish and humans

Infection with local *Leptospira* serogroups and putative serovars in animals and humans was deduced serologically using MAT on blood samples from cattle, rodents, bats, fish and humans. Local *Leptospira* serovars included in the MAT were Sokoine, Kenya, Lora, Canicola and Grippotyphosa, and reference serovars were Hebdomadis, Pomona and Hardjo.

### Ethical consideration

The Ethical Review Board of Sokoine University of Agriculture approved use of animals. The Tanzania Commission for Science and Technology (COSTECH) granted the research permit for use of wild animals (permit no. 2013-260-NA-2014-110). Infected animals were also handled in compliance with the “Animal Research: Reporting In Vivo Experiments” (ARRIVE guidelines) and the Helsinki Declaration [27, 28]. Wild and domestic animal collecting and handling followed the guidelines of the American Society of Mammalogists [23]. Anonymous human participants gave oral consent allowing anonymous screening for leptospirosis and determination of the prevalence. These individuals were those seeking diagnosis of other diseases in the blood at hospitals and abattoir workers.

## Results

### Isolation of leptospires from animals

A total of 52 *Leptospira* isolates were obtained from urine and kidneys of different animal species in Tanzania. The isolation rate of leptospires from different animal hosts ranged from 0.6% in the rodent *Mastomys natalensis* to 8.4% in the African giant pouched rat (*Cricetomys* sp.) (Table 1).

**Table 1:** *Leptospira* isolation rate in different animal species in Tanzania.

Host animal species	Sample cultured	Number of isolates	Percent positive	Reference
African giant pouched rats (*Cricetomys* sp.)	285	24	8.4	This study, [33]
Common field rats (*Mastomys* sp.)	1382	8	0.6	This study
Insectivore shrews (*Crocidura* sp.)	298	11	3.7	This study
Cattle	1021	7	0.68	[17]
Pigs	236	2	0.85	[34]
**Total number of isolates from all animals**			**52**	

### Isolation of leptospires from humans

Four *Leptospira* isolates were obtained from 589 human urine samples that is 0.67% isolation success rate. Eighty-three of the 589 human urine samples were from abattoir workers handling and slaughtering cattle at Morogoro municipal abattoir. Three of the 83 (3.6%) abattoir workers were culture positive. Abattoir workers thus yielded more positive cultures than participants sampled in hospitals (n = 506) from which only one isolate was obtained from an individual living at Kidodi village in the Kilombero river valley that is 150 km away from Morogoro municipality where three isolates were obtained in abattoir workers. The overall isolation rate from both abattoir workers and individuals sampled in hospitals was low (0.19%) with four isolates from 589 individuals. Culturing from fresh 358 blood samples collected in hospitals was negative. The positive cultures from human urine were contaminated and isolates died before carrying out identification.

Common *Leptospira* serovars with their respective animal hosts were: Sokoine (cattle and rodents); Kenya (rodents and shrews); Mwogolo (rodents); Lora (rodents); Qunjian (Canicola) (rodent); serogroup Grippotyphosa (cattle); and an unknown serogroup or serovar from pigs (Table 2), which is not related to common serogroups or serovars currently reported from Tanzania.

**Table 2:** *Leptospira* serovars and animal species from which they were isolated in Tanzania.

*Leptospira* serovars	Serogroup	Cattle	Pigs	Rodent species		Shrew species
				Multimammate rat (*Mastomys* sp)	African giant pouched rat (*Cricetomys* sp.)	*Crocidura s*p.
Sokoine	Icterohaemorrhagiae	**+**			**+**	
Kenya	Ballum			**+**	**+**	**+**
Lora	Australis			**+**		
Grippotyphosa	Grippotyphosa	**+**				
Canicola	Canicola				**+**	
Mwogolo	Icterohaemorrhagiae				**+**	
Unidentified *Leptospira*	Unknown		**+**			

**Fig 1:**
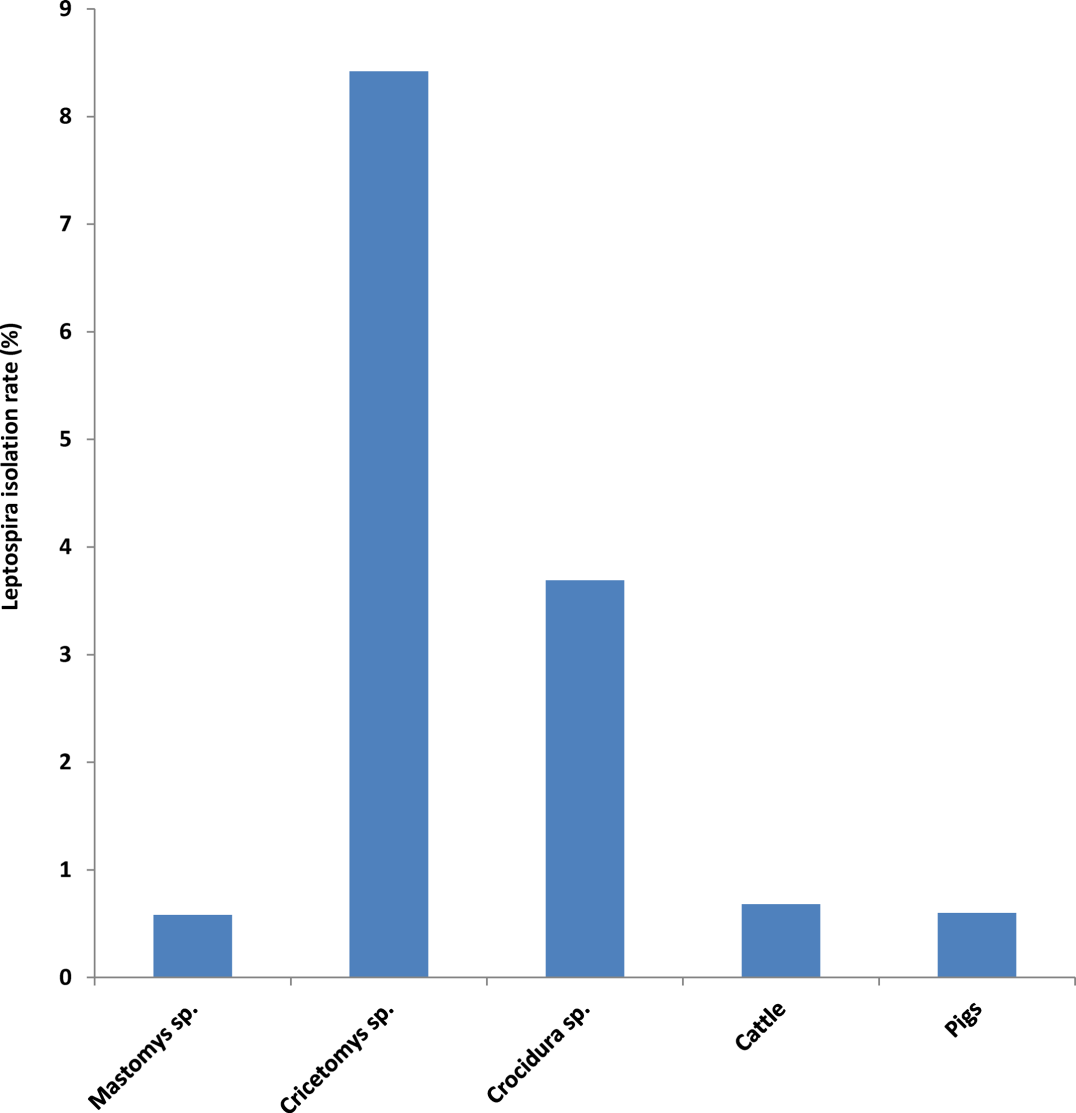
*Leptospira* isolation in various animal hosts in Tanzania. *Cricetomys* sp. and shrews (*Crocidura* sp.) yielded significantly more isolates that other animal species.

The majority of the 52 *Leptospira* isolated obtained in this study were from the African giant pouched rat (*Cricetomys* sp.) which yielded 24 isolates out of 285 specimens (8.42%) and shrews with 11 isolates from 298 specimens (3.7%). Eight isolates were obtained from 1382 *Mastomys natalensis* rats (0.6%), whereas isolation from 1021 cattle and 236 pigs sampled in cattle slaughterhouse and informal pig slaughterhouses yielded 8 isolates (0.68%) and 2 isolates (0.6%), respectively. Three isolates were obtained from *Rattus rattus* but cultures were contaminated before performing and preliminary identification hence they are not presented in this work. No isolates were obtained from *Lemniscomys* spp., *Tatera* spp., *Mus* spp. collected in Morogoro municipality. The pattern of *Leptospira* isolation rates for different animal species is shown in Fig 1.

### Serogrouping

The preliminary serogrouping of the isolates showed that the RM1 isolate reacts with serum for serogroups Canicola, Icterohaemorrhagiae and Sarmin. The titres for serogroup Icterohaemorrhagiae were higher than those of other serogroups (Table 3). Isolates RM4 and RM7 reacted with rabbit serum for serovar Butembo serogroup Autumnalis as well as with serovar Grippotyphosa and Huanuco of serogroup Grippotyphosa, and to some extent, with serovar Djasiman serogroup Djasiman. Isolates coded SH9 and SH25 reacted with rabbit serum for serovars Kenya and Ballum of serogroup Ballum, and serovar Poi serogroup Javanica (Table 3).

**Table 3:** Reaction of selected *Leptospira* isolates with reference antiserum for preliminary assignment of isolates to different *Leptospira* serogroups (titres ≥ 1:20).

S/n	Serogroup	Serovar	Strain	RM1	RM4	RM7	SH9	SH25
1	Australis	Australis	Ballico	-	-	-	-	-
2	Australis	Australis	Jez Bratislava	-	-	-	-	-
3	Autumnalis	Bangkinang	Bangkinang I	-	-	-	-	-
4	Autumnalis	Butembo	Butembo	-	1280	1280	-	-
5	Autumnalis	Carlos	3 C	-	-	-	-	-
6	Autumnalis	Rachmati	Rachmat	-	-	-	-	-
7	Ballum	Ballum	Mus 127	-	-	-	1280	640
8	Ballum	Kenya	Njenga	-	-	-	1280	1280
9	Bataviae	Bataviae	Swart	-	-	-	20	-
10	Canicola	Canicola	Hond Utrecht IV	160	-	-	-	-
11	Canicola	Schueffneri	VI. 90 C	-	-	-	-	-
12	Celledoni	Celledoni	Celledoni	-	-	-	-	-
13	Cynopteri	Cynopteri	3522 C	-	-	-	-	-
14	Djasiman	Djasiman	Djasiman	-	80	160	-	-
15	Grippotyphosa	Grippotyphosa	Moskva V	-	1280	1280	-	-
16	Grippotyphosa	Huanuco	M 4	-	320	640	-	-
17	Hebdomadis	Hebdomadis	Hebdomadis	-	-	-	-	-
18	Hebdomadis	Worfoldi	Worsfold	-	-	-	-	-
19	Icterohaemorrhagiae	Copenhageni	M 20	640	-	-	-	-
20	Icterohaemorrhagiae	Icterohaemorrhagiae	RGA	640	-	-	-	-
21	Javanica	Poi	Poi	-	-	-	320	320
22	Louisiana	Louisiana	LSU 1945	-	-	-	-	-
23	Manhao	Manhao	L 60	-	-	-	-	-
24	Mini	Mini	Sari	-	-	-	-	-
25	Panama	Panama	CZ 214 K	-	-	-	-	-
26	Pomona	Pomona	Pomona	-	-	-	-	-
27	Pyrogenes	pyrogenes	Salinem	-	-	-	-	-
28	Sarmin	Rio	Rr 5	320	-	-	-	-
29	Sarmin	Weaveri	CZ 390	640	-	-	-	-
30	Sejroe	Hardjo	Hardjoprajitno	-	-	-	-	-
31	Sejroe	saxkoebing	Mus 24	-	-	-	-	-
32	Shermani	shermani	1342 K	-	-	-	-	-
33	Tarassovi	Bakeri	LT 79	-	-	-	-	-
34	Tarassovi	Mogden	Compton	-	-	-	-	-
35	Tarassovi	Rama	316	-	-	-	-	-
36	Tarassovi	tarassovi	Perepelicin	-	-	-	-	-
37	Semaranga	Patoc	Patoc I	-	-	-	-	-
38	Fainei	hurstbridge	Hurstbridge	-	-	-	-	-
39	Leptonema	Illini	3055	-	-		-	-
40	Ranarum	Ranarum	ICF	20	-	-	320	160
41	Genomospecies 1	sichuani	79601	-	-	-	-	-
42	Difco contaminant			-	-	-	-	-
43	Lyme	Lyme	10—ATCC3289	-	-	-	-	-

RM1 = serovar Sokoine, RM4 and RM7 = serogroup Grippotyphosa, SH9 and SH25 = serovar Kenya

### Serotyping with monoclonal antibodies

**Fig 2:**
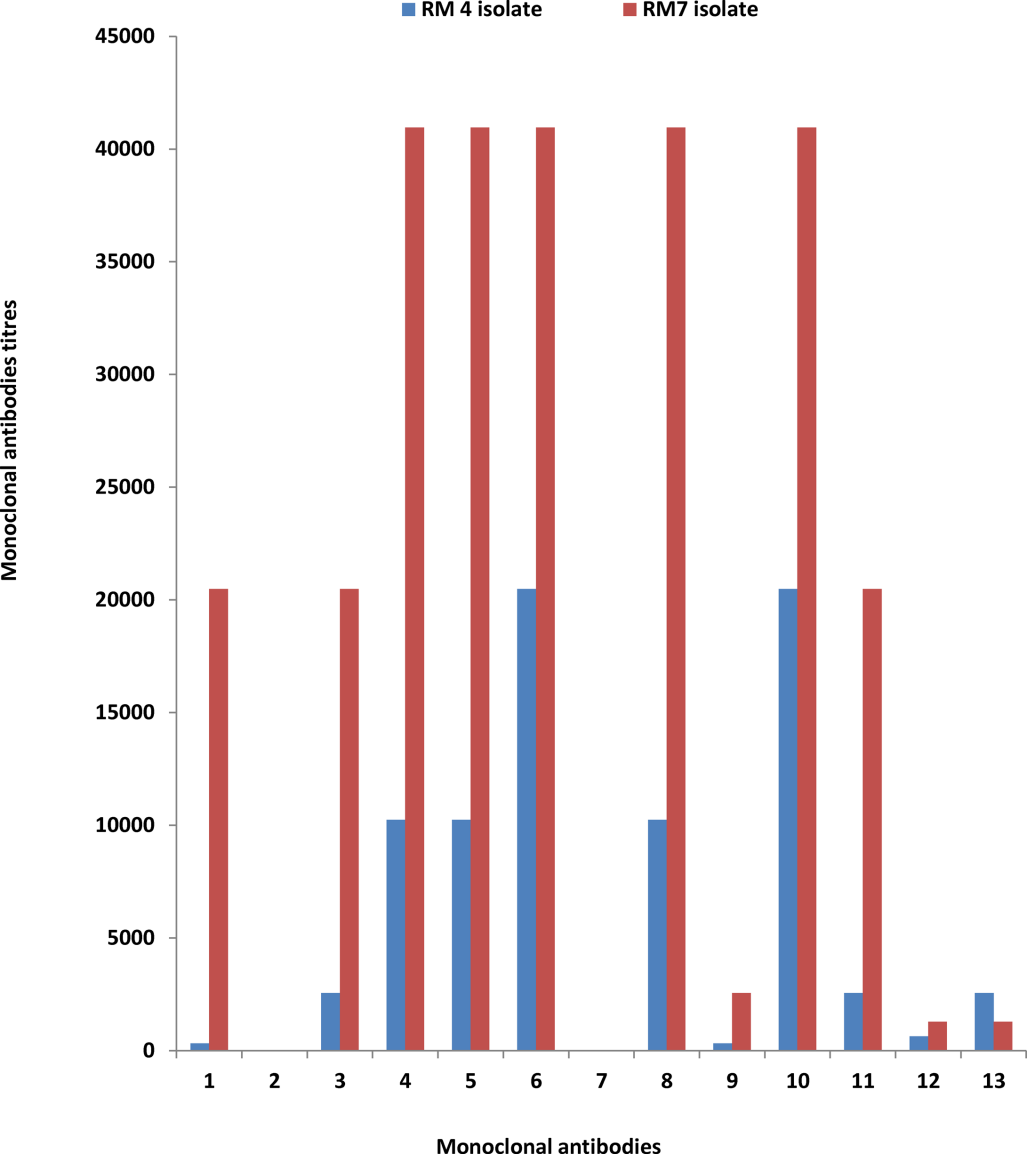
*Leptospira* isolates RM4 and RM7 agglutination with monoclonal antibodies for serogroup Grippotyphosa. 11 of 13 monoclonal antibodies reacted with these isolates.

*Leptospira* isolates assigned to tentative serogroups in preliminary serogrouping with reference rabbit serum were further analysed using specific monoclonal antibodies for those serogroups. Isolates coded RM4 and RM7 did not react with monoclonal antibodies for serogroup Autumnalis, virtually excluding that they belonged to this serogroup. However, they reacted with 11 of the 13 monoclonal antibodies for Grippotyphosa (Fig 2), supporting that they belong to this serogroup (Table 4). Reaction profiles of RM4 and RM7 were most comparable with that of reference *Leptospira* serovar Grippotyphosa serogroup Grippotyphosa.

**Table 4:** Microscopic agglutination titres of isolate RM4 and RM7 reaction with monoclonal antibodies for serogroup Grippotyphosa.

S/n	Monoclonal antibody code	RM4 isolate	RM7 isolate
1	F71C2-4	1: 320	1: 20480
2	F71C3-3	1: 10	1: 10
3	F71C9-4	1: 2560	1: 20480
4	F71C13-4	1: 10240	1: 40960
5	F71C16-6	1: 10240	1: 40960
6	F71C17-5	1: 20480	1: 40960
7	F164C1-1	1: 10	1: 10
8	F165C1-4	1: 10240	1: 40960
9	F165C2-1	1: 320	1: 2560
10	F165C3-4	1: 20480	1: 40960
11	F165C7-5	1: 2560	1: 20480
12	F165C8-3	1: 640	1: 1280
13	F165C12-4	1: 2560	1: 1280

### Distribution and seroprevalence of *Leptospira* serovars in different wild and domestic mammals, fish and humans

Serological tests involving eight commonly occurring *Leptospira* serovars (Table 5) in the Morogoro region revealed variations in occurrence and prevalence of *Leptospira* serovars in wild and domestic mammals, fish and humans. Some *Leptospira* serovars were found often in more than one animal species whereas other serovars were detected in relatively few animal species. Serovars found in different animal species were serovar Kenya and serovar Sokoine detected in domestic mammals, rodents, bats, fish and humans (Table 5).

**Table 5:** Occurrence, distribution and seroprevalence of local and reference *Leptospira* serovars in blood collected from humans, domestic and wild mammals and fish in Tanzania (titres ≥ 1:20).

Hosts	Seroprevalence of *Leptospira* serovars (percentage)							
	Sokoine	Kenya	Lora	Grippotyphosa	Hebdomadis	Pomona	Hardjo	Canicola
Humans (n = 400) [35]	10.75	0.5	NA	0.5	NA	1	3	1.75
Goats and sheep (n = 100)	38	34	NA	14	NA	9	24	9
Pigs (n = 100)	41	27	NA	22	NA	6	26	6
Dogs (n = 100)	39	26	NA	10	NA	9	9	5
Cats (n = 64)	14.1	10.9	NA	7.8	NA	1.6	9.4	1.6
Small rodents (n = 500; 90*)	5–16.9*	2.2*	8.9*	2	1.1*	0.2	0	2.2*–2.8
African giant rats (n = 65)	15.38	NA	NA	1.53	NA	0	NA	13.84
Shrew (*Crocidura* sp.) (n = 4)	25	NA	NA	0	NA	0	NA	0
Bats [20]	19.4	2.8	2.8	NA	0	0	NA	0
Fish [21]	25	29.2		NA	0	6.3	NA	NA

* Leptospirosis prevalence in rodents from study site located 30 km away from *Leptospira* isolation sites in Tanzania; NA-untested

**Fig 3:**
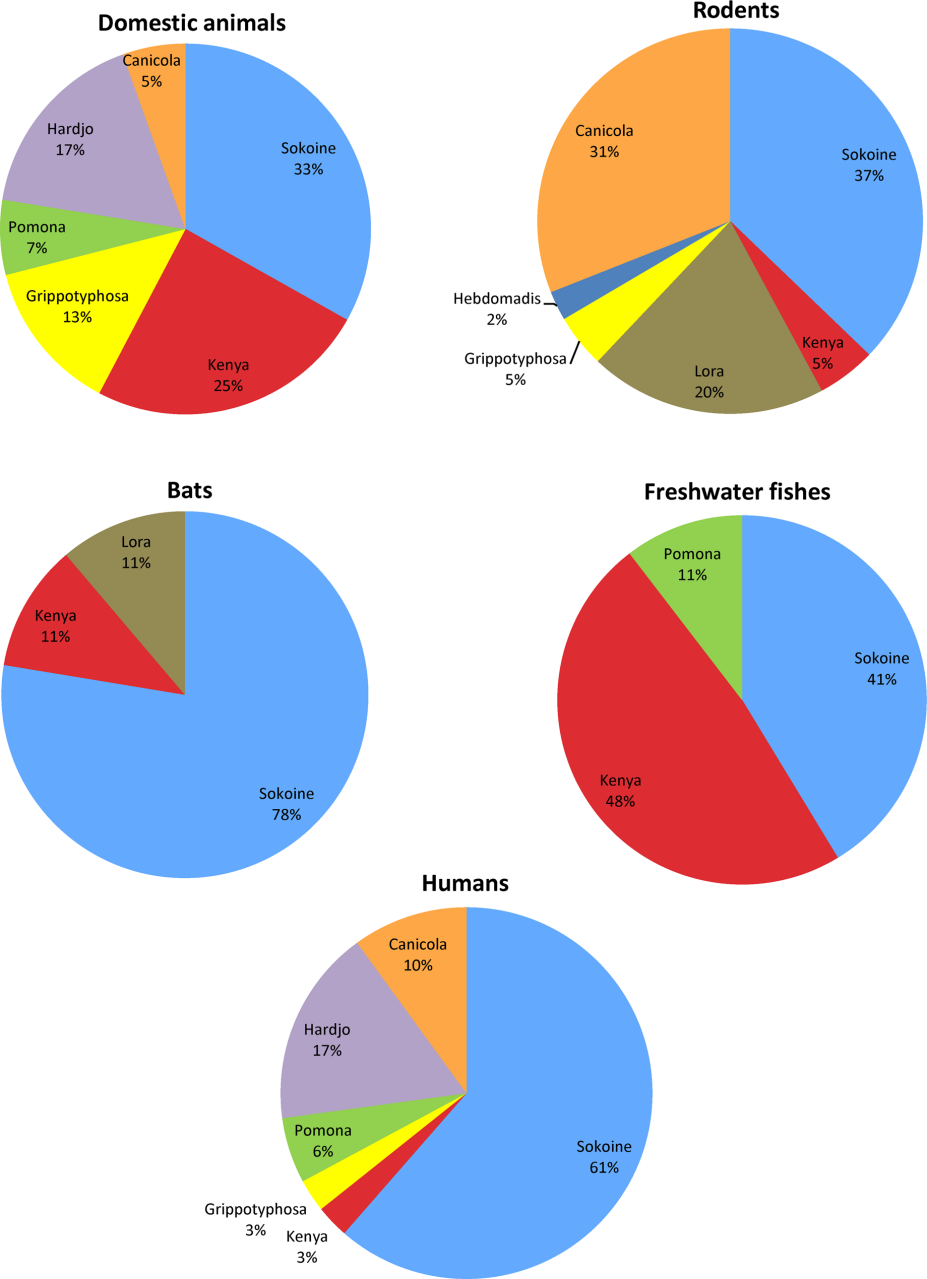
Proportion of prevalence of *Leptospira* serovars in humans, animals and fish.

The seroprevalence of different *Leptospira* serovars in different animal species shows varying seropositivity which indicate that these serovars can be used in subsequent MAT for diagnosis of leptospirosis in animal species reported hereunder (Fig 3). An over 10-fold increase in seroprevalence of leptospirosis in humans, wild and domestic mammals in Tanzania was observed following the use of local serovars such as serovar Sokoine in MAT compared to the reference serovar Icterohaemorrhagiae antigen used in the first study of leptospirosis in animals and humans in Tanzania [17]. These observed increases in rodents went from 1.9% to 16.9%, in humans from 0.26% to 10.75% and in dogs from 37% to 39%. There was also an increase in seroprevalence in MAT with the local Grippotyphosa antigen compared to the reference serovar Grippotyphosa as follows: in rodents from 0% to 2%, in dogs from 0% to 10%, and slight increase was observed in humans from 0.26% to 0.5%. Use of the local serovar Kenya in the MAT of goats, sheep, pigs and dogs also yielded higher prevalence (Table 4), which unfortunately lack comparison since the reference serovar Kenya was not tested in Tanzanian animals.

Fifty-three (75.7%) of the 70 seropositive humans and 31 (62%) of the 50 seropositive animals had lower antibody levels (1:20–1:80) against eight tested *Leptospira* serovars. These titres are below the cut-off point of 1:160 adopted from European setting which has been in use in Tanzania for past two decades due to absence of intensive leptospirosis studies determining cut-off point for Africa. Serovar Sokoine was the most reacting serovar in both humans and animals and contributed to 62.3% (33 out of 53) and 38.7% (12 out of 31) of the seropositivity with lower titres. Majority of the higher titres in 10 out of 17 humans (58.8%) and 13 out of 19 (68.4%) rodents was due to serovar Sokoine. This was followed by serovar Hardjo that contributed to 15% of lower titres and 23.5% (4 out of 17) of the higher titres in humans. Serovar Qunjian **(**Canicola) contributed to 29% (9 out of 31) lower titres and 21% (4 out of 19) of the higher titres in rodents. Serovar Grippotyphosa also contributed to 29% (9 out 31) lower titres in rodents and 5% (1 out of 19) of the higher titres in these animals.

### *Leptospira* serovars for use in MAT in African region

The following *Leptospira* serovars (Table 6) belonging to different species and serogroups yields high positivity when used in MAT of humans and broad range of animal species. Although not exhaustive the listed representative serovars cross reacts with at least 10 other serovars previously reported in central and eastern Africa, Indian ocean islands located near eastern Africa coast, and elsewhere. For example, serovar Sokoine that is widespread in various domestic and wild animal species in Tanzania (Table 5) reacts with serovars Mwogolo, Ndahambukuje and Ndambari found in Congo DRC in central Africa [9], and reacts also with serovars Copenhageni and Icterohaemorrhagiae all belonging to serogroup Icterohaemorrhagiae. Serovar Sokoine again reacts with serovars Weaveri and Rio of serogroup Sarmin as demonstrated in MAT for preliminary serogrouping (Table 3). This cross reactivity broadens detection of leptospirosis caused by these serovars in MAT using serovar Sokoine. Serovar Kenya widely found in rodents in Tanzania was first described in neighbouring Kenya [10]. Its wide distribution in eastern Africa region makes it a good candidate for inclusion in MAT in this region. Serovar Grippotyphosa isolated in cattle in Tanzania cross-reacts with serovar Butembo found in Congo DRC in central Africa which widens the geographic region that this serovar can be applied in MAT. Isolation of additional serovars from Africa is much needed to establish a comprehensive region-specific *Leptospira* serovars for successful leptospirosis diagnosis in African region.

**Table 6:** Local *Leptospira* serovars recommended for use in microscopic agglutination test (MAT) in eastern and central African region.

S/n	Serovar	Cross reacting serovars *
1	Sokoine	Mwogolo
		Ndahambukuje
		Ndambari
		Icterohaemorrhagiae
		Copenhageni
2	Kenya	Peru
		Ballum
		Poi
3	Grippotyphosa	Butembo
		Huanuco
		Djasiman
4	Lora	
5	Canicola	
6	Pig isolate 1	
7	Pig isolate 2	
8	Hardjo	
9	Hebdomadis	
10	Pomona	

* Infecting *Leptospira* serovars detected in MAT using known local serovars and two unknown pig isolates.

The proportion of prevalence of the *Leptospira* serovars recommended for diagnosis of leptospirosis in humans and different animal species is indicated in Fig 3. *Leptospira* serovar Pomona was absent in African giant rats, shrews (*Crocidura* spp.) and bats. Serovar Hebdomadis was not detected in bats and fish whereas Canicola was negative in shrews (*Crocidura* spp.) and bats. Serovar Hardjo was negative in rodent species and Grippotyphosa was not detected in shrew species. *Leptospira* serovars, which did no react with tested animal species, are not presented in Fig 3 below.

## Discussion

This study shows that diverse *Leptospira* serovars occur in a wide range of wild and domestic mammal species, fish and humans in Tanzania. The local *Leptospira* serovar Sokoine and serovar Kenya were the predominant serovars found in many vertebrate species, including fish and humans. Serovar Sokoine for example has been isolated from cattle, rodents (*Mastomys natalensis* and *Cricetomys* sp.) and shrews (*Crocidura* sp.). This serovar has also been detected serologically in humans, cattle, rodents, shrews, bats and fish [20, 21]. The distribution pattern of *Leptospira* in different animals species determined by their isolation rate and seroprevalence indicates that certain rodent species harbour leptospires more often than other species. For example, *Mastomys natalensis*, which are the most abundant rodent species in sub-Saharan Africa [36] yielded few *Leptospira* isolates and had low seroprevalence compared to the African giant pouched rat (*Cricetomys* sp.) and shrews (*Crocidura* sp.), which are also widely distributed across Africa. This lower infection rate in *Mastomys natalensis* could be due to differences in habitats where these species are found, differences in foraging behaviours and/or physiology. For example, *Cricetomys* sp. lives in burrows, which in urban areas may extend to pit latrines and garbage collection areas where they forage. These habitats potentially increase the chances of contracting leptospires and may explain the high isolation rates observed in *Cricetomys* sp. and *Crocidura* sp. (Fig 1). Similarly, *Crocidura* species often appear to live in moist areas. Shrews feed on insects found on soils and wet or moist environments which is reported to support the survival of leptospires for relatively longer periods of time [37, 38]. On the other hand, *Mastomys natalensis* habitats are relatively different from those of *Cricetomys* sp. and *Crocidura* sp. and is considered a peridomestic rodent found in both wild habitats such as shrubs and grasslands as well as crop fields and houses [39]. This study indicates that *Cricetomys* sp. and *Crocidura* sp. serve as major reservoirs of leptospirosis among small mammal species. Future studies aiming at isolation of leptospires to determine serovar prevalence in neglected regions should target the giant African pouched rats (*Cricetomys* sp.) and shrew species (*Crocidura* sp.) or other animal species with similar life styles, e.g. *Rattus norvegicus*.

This study shows that certain *Leptospira* serovars are specific to certain animal species but that some serovars such as Sokoine and Kenya are found in a broad range of animal species including domestic animals, rodents, bats and fish. *Leptospira* isolates belonging to serovars Sokoine and Kenya were serologically detected by MAT in diverse animal species including cattle, sheep, goats, dogs, cats, rodents, bats, fish and humans (Table 5), (Fig 3). Serovar Sokoine belongs to serogroup Icterohaemorrhagiae that majority of its serovars are often reported in humans worldwide [9]. Furthermore, serovars Sokoine and Kenya belong to the *L*. *kirschneri* species that is considered predominant in the East and Central African region including Indian Ocean islands near the East African coast [9, 13]. Other *Leptospira* serovars encountered in animals and humans include serovars Hebdomadis, Hardjo, Pomona, Lora, Australis and Canicola. The seroprevalence of these serovars in different animal species and humans shows relatively lower prevalence compared to the proportions of serovar Sokoine, Kenya and Lora. The observed occurrence and prevalence of these serovars in animals and humans indicate that they are major candidate serovars for inclusion and use as antigens in subsequent MAT for serodiagnosis of leptospirosis in animals and humans in this region. Studies focusing in isolation of new *Leptospira* serovars in humans and animals in Africa are urgently needed to generate a panel of region-specific *Leptospira* serovars for MAT in African continent. In this study inclusion of local serovars, particularly serovar Sokoine, in MAT detection of leptospiral antibodies in rodents and humans in Tanzania revealed an over 10-fold increase in leptospirosis prevalence which was higher than the prevalence reported in the first broad study of leptospirosis in Tanzania [17]. An increase in seroprevalence was also observed following use of the local serovar Grippotyphosa in determination of leptospirosis in humans and animals. Similarly, the local serovar Kenya also demonstrated higher leptospirosis prevalence in goats, sheep, pigs and dogs exceeding those of reference serovars Hebdomadis, Pomona, Hardjo and Canicola (Table 5). This further indicates the necessity for isolation of local *Leptospira* for use in MAT.

Majority of seropositive humans and animals particularly rodents had lower titres against the tested serovars whereas serovar Sokoine and Hardjo had the highest number of individuals with lower and higher titres in humans. Serovar Sokoine had also the highest number of animals with lower and higher titres followed by serovar Canicola. Serovar Grippotyphosa had also higher number of animals with lower titres. Serovar Hardjo was not detected in rodents. These findings indicate the need for establishing the cut-off point for serological diagnosis (MAT) of leptospirosis in humans and animals in Africa where the disease in endemic due to abundance and diversity of reservoir hosts in Africa. Emphasize is needed to isolate and determine antibody levels using animals’ seropositivity and titres, which are common in animals yielding positive isolates.

The findings suggest potential risk of human infection from animal leptospirosis indicated by isolation of three *Leptospira* isolates from urine of three abattoir workers out of 83 individuals (3.6%) sampled among people involved with slaughtering of cattle at Morogoro abattoir where 12 *Leptospira* isolates belonging to serovar Sokoine, Grippotyphosa and Qunjian (Canicola) were isolated from cattle and rodents captured around the abattoir. *Leptospira* isolation success of 3.6% in abattoir workers indicates higher infection risk in this population than in the general population in which only one sample was culture positive (0.19%) out of 506 urine samples collected from patients taking blood tests in hospitals. Indeed the overall rate of *Leptospira* isolation from abattoir workers and other participants from hospitals was lower (0.67%) compared to 3.6% positivity observed in abattoir workers. Abattoir workers rarely ware adequate personal protective gears that can prevent direct contact with animals’ blood and urines when working at this abattoir and other two informal pig slaughterhouses where leptospires were isolated from pigs also in Morogoro. Successful isolation of leptospires from rodents captured inside and around houses which increases the risk of human infections. The observed lower antibody levels in humans may also indicate consistent exposure to leptospires. Isolation of leptospires from a patient at Kidodi village located 150 km away from Morogoro town further indicates high risk of leptospirosis although the identification of the four human isolates was not successful as they died from contamination before preliminary identification. Kidodi village is located along the Kilombero valley where sugarcane farming is the main agricultural activity that is associated with *Leptospira* infection.

Two *Leptospira* isolates obtained from pigs appear to differ from serovars belonging to serogroups Grippotyphosa, Icterohaemorrhagiae and Ballum. The two isolates from pigs did not react with any of the reference rabbit serum for serovars belonging to these serogroups including serum against the most common *Leptospira* serovars Sokoine and Kenya. Further identification of these isolates is required to understand their taxonomic status for serodiagnostic and epidemiological purposes. Furthermore, there is an urgent need for conducting leptospirosis studies focusing on isolation and identification of leptospires from a wide range of terrestrial and aquatic animal species in understudied areas of the African continent. Such studies may enhance mapping of the distribution pattern of *Leptospira* serovars and the actual burden of this disease across Africa. This could be achieved by establishing *Leptospira* isolation facilities in microbiological laboratories, especially in universities and research institutions existing in different African countries. Successful isolation should be followed by preliminary identification of the isolates to at least genus level using darkfield microscopy. Subsequently, the isolates could be identified to serogroup level by MAT as shown in this report (Table 3). The new isolates should also be inoculated into rabbits to produce anti-leptospira antibodies for use in serological typing and MAT [26]. These experimental steps are feasible in most African countries. Further identification of isolates could also be achieved using molecular DNA fingerprinting methods [30, 31] which can enhance comparisons of DNA fingerprint patterns of known serovars with unknown isolates from similar localities prior to the gold standard cross agglutination absorption test (CAAT) recommended for taxonomy of the leptospires [40].

In conclusion, diverse *Leptospira* serovars occur in animals and humans in Tanzania. These *Leptospira* serovars have implications in serodiagnosis of this zoonotic disease in both animals and humans. Serovars Sokoine, Kenya, Grippotyphosa, Lora, Pomona, Hardjo, Hebdomadis and Canicola should be included in leptospirosis diagnosis in Tanzania and neighbouring countries. Further studies focusing on isolation and identification of leptospires from terrestrial and aquatic animal species as well as humans are needed to understand potential circulating serovars in neglected regions where the disease is not recognised and not routinely diagnosed in hospitals. A recent study of causes of fevers among infants and children in northern Tanzania shows that 7.7% were due to leptospirosis [41]. This prevalence could increase when local *Leptospira* serovar Sokoine is used as antigen as has been demonstrated in humans from Morogoro region where initial surveillance conducted in 1996 using reference serovars showed lower prevalence (0.26%) [17], and subsequence surveillance conducted in 2006 using local serovar Sokoine showed higher prevalence (10.7%) [35]. Similar trend was recently observed in rodents whereby previous studies using reference serovar Icterohaemorrhagiae belonging to serogroup Icterohaemorrhagie of which serovar Sokoine also belongs was 5% [17] and a higher prevalence (16.9%) was obtained when local *Leptospira* serovar Sokoine was used in rodent population located 30 km away from where serovar Sokoine was isolated in Morogoro, Tanzania [42]. Generally, this study shows that use of local serovar Sokoine and Kenya in MAT reveals high leptospirosis prevalence in wide range of animal species and calls for reconsideration of previous knowledge that certain *Leptospira* serovars infects only certain animal species. Serovar Sokoine in this case was found in humans, domestic animals (including pet animals), rodents, bats and fishes, hence making it a broad antigen for leptospirosis diagnosis in Africa.

Public awareness of leptospirosis should be promoted to high-risk populations including farmers, livestock keepers, fishermen and abattoir workers. Leptospirosis has been reported also in residents of rural and urban areas interacting with rodents but not engaged with risk occupational activities [43]. Hospital staff, clinicians, veterinarians and policy makers’ needs awareness of existence and high prevalence of leptospirosis in Africa and should consider its diagnosis and treatment. For example in Tanzania and neighbouring countries, diagnosis of leptospirosis in hospitals can be achieved in collaboration with the leptospirosis laboratory at Sokoine University of Agriculture. Capacity building training on leptospirosis diagnosis, *Leptospira* isolation and maintenance of *Leptospira* cultures for use as live antigens for leptospirosis diagnosis should be emphasized in Africa in order to successful control this debilitating and fatal neglected disease.

## Supporting Information

S1 Checklist STARD checklist. (DOC)

S1 Flowchart Flowchart of leptospirosis study in humans. (DOC)

S2 Flowchart Flowchart of leptospirosis study in rodents and shrews. (DOC)

S3 Flowchart Flowchart of leptospirosis study in domestic animals. (DOC)
