# Supplementary material for: Leptospira Serovars for Diagnosis of Leptospirosis in Humans and Animals in Africa: Common Leptospira Isolates and Reservoir Hosts
Source: PLoS Negl Trop Dis. 2015 Dec 1;9(12):e0004251. doi: 10.1371/journal.pntd.0004251 (PMC4666418; doi:10.1371/journal.pntd.0004251)
Supplement: S1 Flowchart — (DOC) [file pntd.0004251.s002.doc]

400 patients randomly sampled in hospitals

All 400 samples subjected to microscopic agglutination test using 6 Leptospira serovars

MAT positive with titre ≥ 1:20 for

– Sokoine (43 samples)

– Grippotyphosa (2 samples)

– Hardjo (12 samples)

– Pomona (4 samples)

– Canicola (7 samples)

– Kenya (2 samples)

Total positive = 70 (17.5%)

MAT negative < 1:20 for serovars:

– Sokoine (357 samples)

– Grippotyphosa (398 samples)

– Hardjo (388 samples)

– Pomona (396 samples)

– Canicola (393 samples)

– Kenya (398 samples)

MAT positive with serovar Sokoine as standard local antigen

Positive ≥ 1:20 = 43 (10.75%)

MAT negative for serovar Sokoine as standard local antigen

< 1:20 = 357 (89.25%)

**S1.** Flow chart of leptospirosis study in humans
