# Supplementary material for: Leptospira Serovars for Diagnosis of Leptospirosis in Humans and Animals in Africa: Common Leptospira Isolates and Reservoir Hosts
Source: PLoS Negl Trop Dis. 2015 Dec 1;9(12):e0004251. doi: 10.1371/journal.pntd.0004251 (PMC4666418; doi:10.1371/journal.pntd.0004251)
Supplement: S2 Flowchart — (DOC) [file pntd.0004251.s003.doc]

500 rodents and shrews captured in wild and houses

All 500 samples tested by microscopic agglutination test (MAT) using 6 Leptospira serovars

MAT positive with titre ≥ 1:20 for

– Sokoine (25 samples)

– Grippotyphosa (10 samples)

– Hardjo (no sample)

– Pomona (1 sample)

– Canicola (14 samples)

– Kenya (no sample)

Total positive = 50 (10%)

MAT negative with titre < 1:20 for serovars:

– Sokoine (475 samples)

– Grippotyphosa (490 samples)

– Hardjo (500 samples)

– Pomona (499 samples)

– Canicola (486 samples)

– Kenya (500 sample)

MAT negative with serovar Sokoine as standard local antigen

< 1:20 = 475 (95%)

MAT positive with serovar Sokoine as standard local antigen

Positive ≥ 1:20 = 25 (5%)*

* A prevalence of 16.9% reported in another study from Tanzania [41]

**S2:** Flow chart of leptospirosis study in rodents and shrews
