# Supplementary material for: Leptospira Serovars for Diagnosis of Leptospirosis in Humans and Animals in Africa: Common Leptospira Isolates and Reservoir Hosts
Source: PLoS Negl Trop Dis. 2015 Dec 1;9(12):e0004251. doi: 10.1371/journal.pntd.0004251 (PMC4666418; doi:10.1371/journal.pntd.0004251)
Supplement: S3 Flowchart — (DOC) [file pntd.0004251.s004.doc]

364 domestic animals animals consisting of dogs, cats, sheep and goats

364 domestic animal serum samples tested by microscopic agglutination test using 6 live Leptospira serovars

MAT positive with titre ≥ 1:20

– Sokoine (127 samples)

– Grippotyphosa (51 samples)

– Hardjo (65 samples)

– Pomona (25 samples)

– Canicola (21 samples)

– Kenya (94 samples)

MAT negative < 1:20 for serovars:

– Sokoine (237 samples)

– Grippotyphosa (313 samples)

– Hardjo (299 samples)

– Pomona (339 samples)

– Canicola (343 samples)

– Kenya (270 samples)

MAT positive with Sokoine the most reactive local antigen

Positive ≥ 1:20 = 127 (34.8%)

MAT negative for serovar Sokoine, the local antigen with titres < 1:20

= 237 (65.1%)

**S3**: Flow chart of leptospirosis study in domestic animals
